# Uncertainty principles for coupled fractional Wigner–Ville distribution

**DOI:** 10.1098/rsos.231579

**Published:** 2024-05-01

**Authors:** Andi Tenri Ajeng Nur, Mawardi Bahri, Nasrullah Bachtiar, Amran Rahim

**Affiliations:** ^1^ Department of Mathematics, Hasanuddin University, Makassar 90245, Indonesia

**Keywords:** coupled fractional Fourier transform, uncertainty principle, fractional Fourier transform

## Abstract

The coupled fractional Wigner–Ville distribution is a more general version of the fractional Wigner–Ville distribution. Main properties including boundedness, Moyal’s formula and inversion formula are studied in detail for the transformation. Additionally, the relation of the coupled fractional Wigner–Ville distribution with the two-dimensional Fourier transform is studied. We also present the relationship between the coupled fractional Wigner–Ville distribution with the two-dimensional Wigner–Ville distribution. We show how the properties and relations allow us to derive several versions of the uncertainty inequalities related to the coupled fractional Wigner–Ville distribution.

## 1. Introduction

In a series of articles [1–8], the fractional Fourier transform has become a standard mathematical tool in a large number of areas including quantum mechanics, neural networks, differential equations, optics, pattern recognition, radar, sonar, and other communication systems. It can be understood as an expansion of the Fourier transform, which was first introduced in 1980 by Namias [9]. In the latest work of the authors [10,11], the coupled fractional Fourier transform has been proposed. The generalized version can be considered as a variant of the two-dimensional fractional Fourier transform. Based on the kernel of the coupled fractional Fourier transform, the authors [12] have constructed the short-time coupled fractional Fourier transform. Later in [13], the authors proposed the coupled fractional Wigner–Ville distribution (CFrWVD), which is a natural generalization of the fractional Wigner–Ville distribution [14] and the classical Wigner–Ville distribution.

Though several essential properties of this generalized transformation have been investigated in detail [13], and the uncertainty principles were also reported [15], several uncertainty principles associated with this transformation such as sharp Hausdorff–Young inequality do not seem to have been realized so far. In contrast, the proof of the uncertainty principles is based on the definition and properties of the CFrWVD, and we implement the relation between the CFrWVD and two-dimensional Fourier transform, the proof of which is simpler. Furthermore, our main results may be viewed as a continuation of the results in [15].

In the present study, we deal with the CFrWVD. Our main contribution is to explore several versions of the uncertainty inequalities concerning the CFrWVD, which is one of the fundamental results related to the transformation. To arrive at the results, we introduce a definition of the CFrWVD and investigate the main properties. We also provide a direct connection between the CFrWVD and the two-dimensional Fourier transform.

The organization of the work is as follows. In §2, we collect some essential facts on the fractional Fourier transform and the coupled fractional Fourier transform. Section 3 concentrates on the derivation of the main properties of the CFrWVD. We also demonstrate its relation with the two-dimensional Fourier transform, which will be useful to obtain some inequalities related to the CFrWVD. Section 4 is devoted to the derivation of some uncertainty principles concerning the CFrWVD. Lastly, in §5, we conclude.

## 2. Preliminaries

First of all, we recall the basic facts related to the fractional Fourier transform (FrFT) and the coupled fractional Fourier transform and their basic properties, which will be needed in the sequel. We also introduce a definition of the CFrWVD. We begin by recalling the well-known definition below.

**Definition 2.1.** We define the space of measurable functions on ${\mathbb{R}}^{2}$ , such that


(2.1) $$\begin{array}{r} \|f{\|}_{L^{r}(R^{2})}={\left(\int_{\;\;R^{2}}|f\left(\tau \right){|}^{r}\;d\tau \right)}^{1/r}<\infty ,\;1\leq r<\infty . \end{array}$$

Here, $\tau =(\tau_{1},\tau_{2})\in R^{2},d\tau =d\tau_{1}d\tau_{2}.$

Especially, for r→∞ , we get


(2.2) $$\begin{array}{r} \|f{\|}_{L^{\infty }(R^{2})}={ess\sup}_{\tau \in R^{2}}|f(\tau )|. \end{array}$$

Furthermore, if f is continuous, then equation (2.2) changes to


(2.3) $$\begin{array}{cc} {∥f∥}_{L^{\infty }\,\left({\mathbb{R}}^{2}\right)}=\sup_{𝝉\in {\mathbb{R}}^{2}}\left|f\,\left(𝝉\right)\right|. & \end{array}$$

The usual inner product of $L^{2}\,\left({\mathbb{R}}^{2}\right)$ is then defined as


(2.4) $$\langle f,g\rangle_{L^{2}(R^{2})}=\int_{\;\;R^{2}}f(\tau )\bar{g(\tau )}d\tau .$$

Let us now introduce a definition of the two-dimensional FrFT.

**Definition 2.2.** The two-dimensional FrFT with parameter θ is defined for a function $f\in L^{1}\,\left({\mathbb{R}}^{2}\right)$ by [3,8]


(2.5) $$F_{\theta }\{f\}(\eta )=\int_{\;\;R^{2}}f(\tau )K_{\theta }(\eta ,\tau )\;d\tau ,$$

where the kernel function $K_{\theta }\,\left(𝛈,𝛕\right)$ is given by


(2.6) $$\begin{array}{r} K_{\theta }(\eta ,\tau )=\left\{ \begin{array}{ll} A_{\theta }e^{i(|\tau {|}^{2}+|\eta {|}^{2})\frac{\cot\theta }{2}-i\tau \cdot \eta \;\csc\theta }, & \;\theta \neq n\pi \\ \delta (\tau -\eta ), & \;\theta =2n\pi \\ \delta (\tau +\eta ), & \;\theta =(2n+1)\pi ,n\in Z, \end{array}\right. \end{array}$$

for which the Dirac delta function $\delta \,\left(𝛕-𝛈\right)=\delta \,\left(\tau_{1}-\eta_{1}\right)\,\delta \,\left(\tau_{2}-\eta_{2}\right)$ and


(2.7) $$\begin{array}{cc} A_{\theta }=\sqrt{\frac{1-i\,\cot\theta }{2\,\pi }},\bar{A_{\theta }}=\sqrt{\frac{1+i\,\cot\theta }{2\,\pi }}. & \end{array}$$

It is straightforward to verify that the FrFT kernel fulfils the following basic properties:


$$\bar{K_{\theta }(\eta ,\tau )}=K_{-\theta }(\eta ,\tau )$$

and


$$\int_{R^{2}}K_{\theta }(\eta ,\tau )\bar{K_{\theta }(\eta^{\prime },\tau )}\;d\tau =\delta (\eta -\eta^{\prime }),$$

where $\bar{K_{\theta }\,\left(𝜼,𝝉\right)}$ stands for the complex conjugate of $K_{\theta }\,\left(𝜼,𝝉\right)$ .

**Definition 2.3.** Suppose that $f\in L^{1}\,\left({\mathbb{R}}^{2}\right)$ and ${ℱ}_{\theta }\,\left\{f\right\}\in L^{1}\,\left({\mathbb{R}}^{2}\right)$ . The inverse transform of the two-dimensional FrFT of the function f is given by the integral [3,8]


(2.8) $$\begin{array}{rl} f(\tau ) & =F_{\theta }^{-1}[F_{\theta }\{f\}](\tau )\\ & =\int_{\;\;R^{2}}F_{\theta }\{f\}(\eta )\bar{A_{\theta }}e^{-i(|\tau {|}^{2}+|\eta {|}^{2})\frac{\cot\theta }{2}-i\tau \cdot \eta \;\;\csc\theta }\;d\eta . \end{array}$$

Equation (2.8) shows how to recover the function from its FrFT. The following definition is important in this article.

**Definition 2.4.** (CFrFT definition). The coupled fractional Fourier transform (CFrFT) is defined for any function $f\in L^{1}\,\left({\mathbb{R}}^{2}\right)\cap L^{2}\,\left({\mathbb{R}}^{2}\right)$ by [10,11]


(2.9) $$\begin{array}{rl} F_{\alpha ,\beta }\{f\}(\eta ) & =\int_{\;\;R^{2}}f(\tau )K_{\alpha ,\beta }(\eta ,\tau )d\tau \\ & =\int_{\;\;R^{2}}f(\tau )d(\gamma )e^{-i(a(\gamma )(|\tau {|}^{2}+|\eta {|}^{2})-\tau \cdot M\eta )}d\tau . \end{array}$$

In this case,


(2.10) $$K_{\alpha ,\beta }(\eta ,\tau )=d(\Upsilon )e^{-i(\alpha (\Upsilon )(|\tau {|}^{2}+|\eta {|}^{2})-\tau .M\eta )}.$$

Here,


$$\gamma =\frac{\alpha +\beta }{2},\;\delta =\frac{\alpha -\beta }{2},\;a=(\gamma )=\frac{\cot\gamma }{2},\;b=(\gamma ,\delta )=\frac{\cos\delta }{\sin\gamma },\;c(\gamma ,\delta )=\frac{\sin\delta }{\sin\gamma },\;d(\gamma )=\frac{ie^{-i\gamma }}{2\pi \sin\gamma },\;M=\left(\begin{array}{cc} b(\gamma ,\delta ) & c(\gamma ,\delta )\\ -c(\gamma ,\delta ) & b(\gamma ,\delta ) \end{array}\right),$$

with α,β∈R such that α+β∉2πZ.

Equation (2.9) may be expressed in the form


(2.11) $$F_{\alpha ,\beta }\{f\}(\eta )=d(\gamma )\int_{\;\;R^{2}}\left(f(\tau )e^{-ia(\gamma )|\tau {|}^{2}}\right)e^{-ia(\gamma )|\eta {|}^{2}}e^{i\tau \cdot M\eta }d\tau .$$

Denoting


(2.12) $$g\,\left(𝝉\right)=f\,\left(𝝉\right)\,e^{-i\,a\,\left(\gamma \right)\,{\left|𝝉\right|}^{2}},$$

we obtain


|g⁢(𝝉)|=|f⁢(𝝉)|.

Due to equation (2.12), equation (2.11) takes the following form:


(2.13) $$\begin{array}{rl} {\left(d(\gamma )\right)}^{-1}e^{ia(\gamma )|\eta {|}^{2}}F_{\alpha ,\beta }\{f\}(\eta ) & =\int_{\;\;R^{2}}g(\tau )e^{i\tau \cdot M\eta }d\tau \\ & =F\{g\}(-M\eta ). \end{array}$$

This equation describes the basic connection between the CFrFT and the two-dimensional Fourier transform. Here, ℱ⁢{f} stands for the two-dimensional Fourier transform of $f\in L^{2}\,\left({\mathbb{R}}^{2}\right)$ given by [16–18]


(2.14) $$F\{f\}(\eta )=\int_{\;\;R^{2}}f(\tau )e^{-i\tau \cdot \eta }d\tau .$$

The function f⁢(𝝉) in equation (2.14) can be obtained in terms of the CFrFT using the following definition.

**Definition 2.5.** For every $f\in {ℱ}_{\alpha ,\beta }\,\left\{f\right\},L^{1}\,\left({\mathbb{R}}^{2}\right)$ , the inverse of the CFrFT is given by [10,11]


(2.15) $$\begin{array}{rl} f(\tau ) & =\int_{\;\;R^{2}}F_{\alpha ,\beta }\{f\}(\eta )\bar{K_{\alpha ,\beta }(\eta ,\tau )}d\eta \\ & =\bar{d(\gamma )}\int_{\;\;R^{2}}F_{\alpha ,\beta }\{f\}(\eta )e^{i(a(\gamma )(|\tau {|}^{2}+|\eta {|}^{2})-\tau \cdot M\eta )}d\eta . \end{array}$$

A Parseval identity is valid for the CFrFT. For any $f,g\in L^{2}\,\left({\mathbb{R}}^{2}\right)$ , the following relation is satisfied:


(2.16) $$\begin{array}{r} \langle f,g\rangle_{L^{2}(R^{2})}=\langle F_{\alpha ,\beta }\{f\},F_{\alpha ,\beta }\{g\}\rangle_{L^{2}(R^{2})} \end{array}$$

and


(2.17) $${∥f∥}_{L^{2}\,\left({\mathbb{R}}^{2}\right)}^{2}={∥{ℱ}_{\alpha ,\beta }\,\left\{f\right\}∥}_{L^{2}\,\left({\mathbb{R}}^{2}\right)}^{2}.$$

Let us recall a definition of the CFrWVD [13]. It is constructed by replacing the kernel Fourier in the definition of the two-dimensional Wigner–Ville distribution with the kernel function of the CFrFT.

**Definition 2.6.** The CFrWVD is defined for functions $f,g\in L^{2}\,\left({\mathbb{R}}^{2}\right)$ by [13]


(2.18) $$W_{f,g}^{\alpha ,\beta }(x,\eta )=\int_{\;\;R^{2}}f\left(x+\frac{\tau }{2}\right)\bar{\;g\left(x-\frac{\tau }{2}\right)}d(\gamma )e^{-i(a(\gamma )(|\tau {|}^{2}+|\eta {|}^{2})-\tau \cdot M\eta )}d\tau .$$

## 3. Coupled fractional Wigner–Ville distribution and main properties

In this section, we investigate the essential properties of the CFrWVD. The properties will be used in the later part of this article.

From equation (2.18), we get


(3.1) $$\begin{array}{rl} W_{f,g}^{\alpha ,\beta }(x,\eta )= & \int_{\;\;R^{2}}h_{f,g}\left(x,\tau \right)K_{\alpha ,\beta }(\eta ,\tau )d\tau \\ = & F_{\alpha ,\beta }\{h_{f,g}\left(x,\tau \right)\}(\eta ), \end{array}$$

where $K_{\alpha ,\beta }\,\left(𝜼,𝝉\right)$ is defined by equation (2.10) and


(3.2) $$h_{f,g}\left(x,\tau \right)=f\left(x+\frac{\tau }{2}\right)\bar{g\left(x-\frac{\tau }{2}\right)}.$$

Due to equation (2.18), we have


$$W_{f,g}^{\alpha ,\beta }(x,\eta )=\int_{\;\;R^{2}}h_{f,g}\left(x,\tau \right)d(\gamma )e^{-i(a(\gamma )(|\tau {|}^{2}+|\eta {|}^{2})-\tau \cdot M\eta )}d\tau .$$

The above equation may be expressed as


$$d^{-1}(\gamma )e^{ia(\gamma )|\eta {|}^{2}}W_{f,g}^{\alpha ,\beta }(x,\eta )=\int_{\;\;R^{2}}h_{f,g}\left(x,\tau \right)e^{-ia(\gamma )|\tau {|}^{2}}e^{i\tau \cdot M\eta }d\tau .$$

Applying equation (2.14) to the above identity gives


(3.3) $$d^{-1}(\gamma )e^{ia(\gamma )|\eta {|}^{2}}W_{f,g}^{\alpha ,\beta }(x,\eta )=F\left\{{\overset{ˇ}{h}}_{f,g}\left(x,\tau \right)\right\}(-M\eta ).$$

This equation is equal to


(3.4) $$W_{f,g}^{\alpha ,\beta }(x,\eta )=F\left\{{\overset{ˇ}{h}}_{f,g}\left(x,\tau \right)\right\}(-M\eta )d(\gamma )e^{-ia(\gamma )|\eta {|}^{2}},$$

where


(3.5) $${\overset{ˇ}{h}}_{f,g}\left(x,\tau \right)=f\left(x+\frac{\tau }{2}\right)\bar{\;g\left(x-\frac{\tau }{2}\right)}e^{-ia(\gamma )|\tau {|}^{2}}.$$

Equation (3.4) describes a direct interaction between the CFrWVD and the two-dimensional Fourier transform. It plays a crucial for deriving the main results in this article.

Further, from definition 2.6, we have


(3.6) $$\begin{array}{rl} W_{f,g}^{\alpha ,\beta }(x,\eta ) & =\int_{\;\;R^{2}}f\left(x+\frac{\tau }{2}\right)\bar{\;g\left(x-\frac{\tau }{2}\right)}d(\gamma )e^{-i(a(\gamma )(|\tau {|}^{2}+|\eta {|}^{2})-\tau \cdot M\eta )}d\tau \\ & =d(\gamma )e^{-ia(\gamma )|\eta {|}^{2}}\int_{\;\;R^{2}}f\left(x+\frac{\tau }{2}\right)\bar{\;g\left(x-\frac{\tau }{2}\right)}e^{-ia(\gamma )|\tau {|}^{2}}e^{-\tau \cdot M\eta }d\tau \\ & =d(\gamma )e^{-ia(\gamma )|\eta {|}^{2}}W_{\overset{ˇ}{f},g}(x,M\eta ), \end{array}$$

where $\overset{ˇ}{f}\,\left(𝒙+\frac{𝝉}{2}\right)=f\,\left(𝒙+\frac{𝝉}{2}\right)\,e^{-i\,a\,\left(\gamma \right)\,{\left|𝝉\right|}^{2}}$ and $W_{\overset{ˇ}{f},g}\,\left(𝒙,M\,𝜼\right)$ are the two-dimensional Wigner–Ville distribution [16,17]. Equation (3.6) above explains the direct relation of the CFrWVD to the two-dimensional Wigner–Ville distribution.

Some important properties of the CFrWVD above are as follows.

**Theorem 3.1.** (Boundedness). *Let* $f,g\in L^{2}\,\left({\mathbb{R}}^{2}\right)$ *, then we have*


(3.7) $${\left|{𝒲}_{f,g}^{\alpha ,\beta }\,\left(𝒙,𝜼\right)\right|}^{2}\leq \frac{4}{\pi^{2}\,{\left|\sin\gamma \right|}^{2}}\,{∥f∥}_{L^{2}\,\left({\mathbb{R}}^{2}\right)}^{2}\,{∥g∥}_{L^{2}\,\left({\mathbb{R}}^{2}\right)}^{2}.$$

*Proof*. Thanks to the Cauchy–Schwarz inequality, we obtain


$$\begin{array}{rl} {\left|W_{f,g}^{\alpha ,\beta }(x,\eta )\right|}^{2} & =|\int_{\;\;R^{2}}f\left(x+\frac{\tau }{2}\right)\bar{\;g\left(x-\frac{\tau }{2}\right)}d(\gamma )e^{-i(a(\gamma )(|\tau {|}^{2}+|\eta {|}^{2})-\tau \cdot M\eta )}d\tau {|}^{2}\\ & \leq \int_{\;\;R^{2}}|f\left(x+\frac{\tau }{2}\right)\bar{\;g\left(x-\frac{\tau }{2}\right)}d(\gamma ){|}^{2}d\tau \\ & =|d(\gamma ){|}^{2}\int_{\;\;R^{2}}{\left|f\left(x+\frac{\tau }{2}\right)\right|}^{2}d\tau \int_{\;\;R^{2}}|\bar{g(x-\frac{\tau }{2})}{|}^{2}d\tau \\ & =\frac{4}{\pi^{2}|\sin\gamma {|}^{2}}{\left\|f\right\|}_{L^{2}\left(R^{2}\right)}^{2}{\left\|g\right\|}_{L^{2}\left(R^{2}\right)}^{2}. \end{array}$$

This shows that ${𝒲}_{f,g}^{\alpha ,\beta }\,\left(𝒙,𝜼\right)$ is bounded on $L^{2}\,\left({\mathbb{R}}^{2}\right)$ .

**Theorem 3.2.** (Moyal’s formula). *For any functions* $f_{1},f_{2}\in L^{2}\,\left({\mathbb{R}}^{2}\right)$ *and* $g_{1},g_{2}\in L^{2}\,\left({\mathbb{R}}^{2}\right)$ . *Then, one has*


(3.8) $$\int_{\;\;R^{2}}\int_{\;\;R^{2}}W_{f_{1},g_{1}}^{\alpha ,\beta }(x,\eta )\bar{W_{f_{2},g_{2}}^{\alpha ,\beta }(x,\eta )}d\eta dx=4\langle f_{1},f_{2}\rangle_{L^{2}(R^{2})}\langle g_{2},g_{1}\rangle_{L^{2}(R^{2})}.$$

*Proof*. With the aid of equations (2.16), (3.1) and (3.2), we find that


(3.9) $$\begin{array}{rl} & \int_{\;\;R^{2}}\int_{\;\;R^{2}}W_{f_{1},g_{1}}^{\alpha ,\beta }(x,\eta )\bar{W_{f_{2},g_{2}}^{\alpha ,\beta }(x,\eta )}d\eta dx\\ & \;=\int_{\;\;R^{2}}\int_{\;\;R^{2}}F_{\alpha ,\beta }\{h_{f_{1},g_{1}}\left(x,\tau \right)\}(\eta )\bar{F_{\alpha ,\beta }\{h_{f_{2},g_{2}}\left(x,\tau \right)\}(\eta )}d\eta dx\\ & \;=\int_{\;\;R^{2}}\int_{\;\;R^{2}}h_{f_{1},g_{1}}\left(x,\tau \right)\bar{h_{f_{2},g_{2}}\left(x,\tau \right)}d\tau dx\\ & \;=\int_{\;\;R^{2}}\int_{\;\;R^{2}}f_{1}\left(x+\frac{\tau }{2}\right)\bar{\;g_{1}\left(x-\frac{\tau }{2}\right)}\bar{f_{2}\left(x+\frac{\tau }{2}\right)\bar{\;g_{2}\left(x-\frac{\tau }{2}\right)}}d\tau dx\\ & \;=\int_{\;\;R^{2}}\int_{\;\;R^{2}}f_{1}\left(x+\frac{\tau }{2}\right)\bar{\;g_{1}\left(x-\frac{\tau }{2}\right)}\;g_{2}\left(x-\frac{\tau }{2}\right)\bar{f_{2}\left(x+\frac{\tau }{2}\right)}d\tau dx. \end{array}$$

We use Fubini’s theorem to obtain


$$\begin{array}{rl} \int_{\;\;R^{2}}\int_{\;\;R^{2}} & W_{f,g}^{\alpha ,\beta }\{f_{1}\}(x,\eta )\bar{W_{f,g}^{\alpha ,\beta }\{f_{2}\}(x,\eta )}d\eta dx\\ & =\int_{\;\;R^{2}}f_{1}\left(x+\frac{\tau }{2}\right)\bar{f_{2}\left(x+\frac{\tau }{2}\right)}d\tau \int_{\;\;R^{2}}\bar{g_{1}\left(x-\frac{\tau }{2}\right)}\;g_{2}\left(x-\frac{\tau }{2}\right)dx\\ & =4\langle f_{1},f_{2}\rangle_{L^{2}(R^{2})}\langle g_{2},g_{1}\rangle_{L^{2}(R^{2})}, \end{array}$$

which proves equation (3.8).

An immediate consequence of the above theorem is the following:

1. f $g_{1}=g_{2}=g$ then

(3.10) $$\int_{\;\;R^{2}}\int_{\;\;R^{2}}W_{f_{1},g}^{\alpha ,\beta }(\eta ,x)\bar{W_{f_{2},g}^{\alpha ,\beta }(x,\eta )}d\eta dx=4\|g{\|}_{L^{2}(R^{2})}^{2}\langle f_{1},f_{2}\rangle_{L^{2}(R^{2})}.$$

1. If $f_{1}=f_{2}$ and $g_{1}=g_{2}$ then

(3.11) $$\int_{\;\;R^{2}}\int_{\;\;R^{2}}|W_{f_{1},g_{1}}^{\alpha ,\beta }(\eta ,x){|}^{2}d\eta dx=4\|g_{1}{\|}_{L^{2}(R^{2})}^{2}\|f_{1}{\|}_{L^{2}(R^{2})}^{2}.$$

**Theorem 3.3.** (Inversion formula). *Let* $f,g\in L^{2}\,\left({\mathbb{R}}^{2}\right)$ *be two functions. Then, every* $f\in L^{2}\,\left({\mathbb{R}}^{2}\right)$ *we have*


(3.12) $$f\left(\tau \right)=\frac{1}{g\left(0\right)}\int_{\;\;R^{2}}W_{f,g}^{\alpha ,\beta }\left(\frac{\tau }{2},\eta \right)\bar{K_{\alpha ,\beta }(\eta ,\tau )}d\eta .$$

*Proof*. From equations (3.1) and (3.2), we get


(3.13) $$\begin{array}{r} f\left(x+\frac{\tau }{2}\right)\bar{\;g\left(x-\frac{\tau }{2}\right)}=\int_{\;\;R^{2}}W_{f,g}^{\alpha ,\beta }(x,\eta )\bar{K_{\alpha ,\beta }(\eta ,\tau )}d\eta . \end{array}$$

If we put $𝒙=\frac{𝝉}{2}$ , the above identity is turned into


$$\begin{array}{r} f\left(\tau \right)\bar{g\left(0\right)}=\int_{\;\;R^{2}}W_{f,g}^{\alpha ,\beta }\left(\frac{\tau }{2},\eta \right)\bar{K_{\alpha ,\beta }(\eta ,\tau )}d\eta , \end{array}$$

which completes the proof.

**Remark 3.4.** It should be noticed that theorems 3.2 and 3.3 in the present work differ in terms of constants from the ones proposed in [13].

To motivate the need for the CFrWVD mentioned earlier, we will look at the examples below.

**Example 3.5.** Find the CFrWVD of functions f and g , given by


(3.14) $$f\,\left(𝝉\right)=e^{-{\left|𝝉\right|}^{2}}$$

and


(3.15) g⁢(𝝉)={1,for⁢  0≤τ1,τ2≤10,τ1,τ2 elsewhere.

**Solution.** It follows from equation (2.18) that


(3.16) $$\begin{array}{rl} W_{f,g}^{\alpha ,\beta }(x,\eta ) & =d(\gamma )\int_{\;\;R^{2}}e^{-{\left|x+\frac{\tau }{2}\right|}^{2}}g\left(x-\frac{\tau }{2}\right)e^{-i(a(\gamma )(|\tau {|}^{2}+|\eta {|}^{2})-\tau \cdot M\eta )}d\tau \\ & =d(\gamma )\int_{\;\;R^{2}}e^{-\left({\left(x_{1}+\frac{\tau_{1}}{2}\right)}^{2}+{\left(x_{2}+\frac{\tau_{2}}{2}\right)}^{2}\right)}g\left(x-\frac{\tau }{2}\right)\\ & \;\times e^{-i(a(\gamma )(\tau_{1}^{2}+\tau_{2}^{2}+\eta_{1}^{2}+\eta_{2}^{2})-\tau_{1}\left(b\eta_{1}+c\eta_{2}\right)-\tau_{2}\left(b\eta_{2}-c\eta_{1}\right)}d\tau_{1}d\tau_{2}\\ & =d(\gamma )\int_{2(x_{1}-1)}^{2x_{1}}\int_{2(x_{2}-1)}^{2x_{2}}e^{-{\left(x_{1}+\frac{\tau_{1}}{2}\right)}^{2}-ia(\gamma )\tau_{1}^{2}+\tau_{1}(b\eta_{1}+c\eta_{2})}\\ & =d(\gamma )e^{-ia(\gamma )(\eta_{1}^{2}+\eta_{2}^{2})+(x_{1}^{2}+x_{2}^{2})}\int_{2(x_{1}-1)}^{2x_{1}}e^{-\frac{\tau_{1}^{2}}{4}-ia(\gamma )\tau_{1}^{2}+\tau_{1}(b\eta_{1}+c\eta_{2})-\tau_{1}x_{1}}d\tau_{1}\\ & \;\times \int_{2(x_{2}-1)}^{2x_{2}}e^{-\frac{\tau_{2}^{2}}{4}-ia(\gamma )\tau_{2}^{2}+\tau_{2}(b\eta_{2}+c\eta_{1})-\tau_{2}x_{2}}d\tau_{2}. \end{array}$$

Furthermore, we get


(3.17) $$\begin{array}{cc} {𝒲}_{f,g}^{\alpha ,\beta }\,\left(𝒙,𝜼\right) & =d\,\left(\gamma \right)\,e^{-i\,a\,\left(\gamma \right)\,\left(\eta_{1}^{2}+\eta_{2}^{2}\right)+\left(x_{1}^{2}+x_{2}^{2}\right)}\,\int_{2\,\left(x_{1}-1\right)}^{2\,x_{1}}e^{\left(\frac{1}{4}+i\,a\,\left(\gamma \right)\right)\,\tau_{1}^{2}+\tau_{1}\,\left(b\,\eta_{1}+c\,\eta_{2}-x_{1}\right)}\,𝑑\tau_{1}\\ & \times \int_{2\,\left(x_{2}-1\right)}^{2\,x_{2}}e^{\left(\frac{1}{4}+i\,a\,\left(\gamma \right)\right)\,\tau_{2}^{2}+\tau_{2}\,\left(b\,\eta_{2}+c\,\eta_{1}-x_{2}\right)}d\tau_{2}\\ & =d\,\left(\gamma \right)\,e^{-i\,a\,\left(\gamma \right)\,\left(\eta_{1}^{2}+\eta_{2}^{2}\right)+\left(x_{1}^{2}+x_{2}^{2}\right)}\,\int_{2\,\left(x_{1}-1\right)}^{2\,x_{1}}e^{\left(\frac{1}{4}+i\,a\,\left(\gamma \right)\right)\,\left(\tau_{1}^{2}-\tau_{1}\,\frac{b\,\eta_{1}+c\,\eta_{2}-x_{1}}{\frac{1}{4}+i\,a\,\left(\gamma \right)}\right)}\,𝑑\tau_{1}\\ & \times \int_{2\,\left(x_{2}-1\right)}^{2\,x_{2}}e^{\left(\frac{1}{4}+i\,a\,\left(\gamma \right)\right)\,\left(\tau_{2}^{2}-\tau_{2}\,\frac{b\,\eta_{2}+c\,\eta_{1}-x_{2}}{\frac{1}{4}+i\,a\,\left(\gamma \right)}\right)}d\tau_{2}. \end{array}$$

Hence,


(3.18) $$\begin{array}{rl} & W_{f,g}^{\alpha ,\beta }(x,\eta )\\ & =d(\gamma )e^{-ia(\gamma )(\eta_{1}^{2}+\eta_{2}^{2})+(x_{1}^{2}+x_{2}^{2})}\int_{2(x_{1}-1)}^{2x_{1}}e^{\left(\frac{1}{4}+ia(\gamma )\right){\left(\tau_{1}-\frac{\left(b\eta_{1}+c\eta_{2}-x_{1}\right)}{2\left(\frac{1}{4}+ia(\gamma )\right)}\right)}^{2}}e^{-\frac{{\left(b\eta_{1}+c\eta_{2}-x_{1}\right)}^{2}}{4\left(\frac{1}{4}+ia(\gamma )\right)}}d\tau_{1}\\ & \;\times \int_{2(x_{2}-1)}^{2x_{2}}e^{\left(\frac{1}{4}+ia(\gamma )\right){\left(\tau_{2}-\frac{\left(b\eta_{2}+c\eta_{1}-x_{2}\right)}{2\left(\frac{1}{4}+ia(\gamma )\right)}\right)}^{2}}e^{-\frac{{\left(b\eta_{2}+c\eta_{1}-x_{2}\right)}^{2}}{4\left(\frac{1}{4}+ia(\gamma )\right)}}d\tau_{2}\\ & =d(\gamma )e^{-ia(\gamma )(\eta_{1}^{2}+\eta_{2}^{2})+(x_{1}^{2}+x_{2}^{2})-\frac{{\left(b\eta_{1}+c\eta_{2}-x_{1}\right)}^{2}}{1+i4a(\gamma )}-\frac{{\left(b\eta_{2}+c\eta_{1}-x_{2}\right)}^{2}}{1+i4a(\gamma )}}\\ & \;\times \int_{2(x_{1}-1)}^{2x_{1}}e^{-{\left(\sqrt{\frac{1}{4}+ia(\gamma )}\tau_{1}-\frac{\left(b\eta_{1}+c\eta_{2}-x_{1}\right)}{2\sqrt{\frac{1}{4}+ia(\gamma )}}\right)}^{2}}d\tau_{1}\int_{2(x_{2}-1)}^{2x_{2}}e^{-{\left(\sqrt{\frac{1}{4}+ia(\gamma )}\tau_{2}-\frac{\left(b\eta_{2}+c\eta_{1}-x_{2}\right)}{2\sqrt{\frac{1}{4}+ia(\gamma )}}\right)}^{2}}d\tau_{2}. \end{array}$$

If we set


(3.19) $$\begin{array}{r} u=\frac{\left(b\eta_{1}+c\eta_{2}-x_{1}\right)}{2\sqrt{\frac{1}{4}+ia(\gamma )}}-\sqrt{\frac{1}{4}+ia(\gamma )}\tau_{1} \end{array}$$

and


(3.20) $$\begin{array}{cc} z=\frac{\left(b\,\eta_{2}+c\,\eta_{1}-x_{2}\right)}{2\,\sqrt{\frac{1}{4}+i\,a\,\left(\gamma \right)}}-\sqrt{\frac{1}{4}+i\,a\,\left(\gamma \right)}\,\tau_{2}, & \end{array}$$

then we get


(3.21) $$\begin{array}{rl} W_{f,g}^{\alpha ,\beta }(x,\eta ) & =\frac{\pi }{1+i4a(\gamma )}d(\gamma )e^{-ia(\gamma )(\eta_{1}^{2}+\eta_{2}^{2})-(x_{1}^{2}+x_{2}^{2})-\frac{{\left(b\eta_{1}+c\eta_{2}-x_{1}\right)}^{2}}{1+i4a(\gamma )}-\frac{{\left(b\eta_{2}+c\eta_{1}-x_{2}\right)}^{2}}{1+i4a(\gamma )}}\\ & \;[(\mathrm{erf}(\frac{\left(b\eta_{1}+c\eta_{2}-x_{1}\right)}{2\sqrt{\frac{1}{4}+ia(\gamma )}}+2\sqrt{\frac{1}{4}+ia(\gamma )}(x_{1}-1))\\ & \;\;-\mathrm{erf}(\frac{\left(b\eta_{1}+c\eta_{2}-x_{1}\right)}{2\sqrt{\frac{1}{4}+ia(\gamma )}}-2x_{1}\sqrt{\frac{1}{4}+ia(\gamma )}))\\ & \;\times (\mathrm{erf}(\frac{\left(b\eta_{2}+c\eta_{1}-x_{2}\right)}{2\sqrt{\frac{1}{4}+ia(\gamma )}}+2\sqrt{\frac{1}{4}+ia(\gamma )}(x_{2}-1))\\ & \;\;-\mathrm{erf}(\frac{\left(b\eta_{2}+c\eta_{1}-x_{2}\right)}{2\sqrt{\frac{1}{4}+ia(\gamma )}}-2x_{2}\sqrt{\frac{1}{4}+ia(\gamma )}))], \end{array}$$

where


$$\mathrm{erf}z=\frac{2}{\sqrt{\pi }}\,\int_{0}^{z}e^{-t^{2}}\,𝑑t,\mathrm{for}\,\mathrm{all}\,z.$$

**Figure 1.**
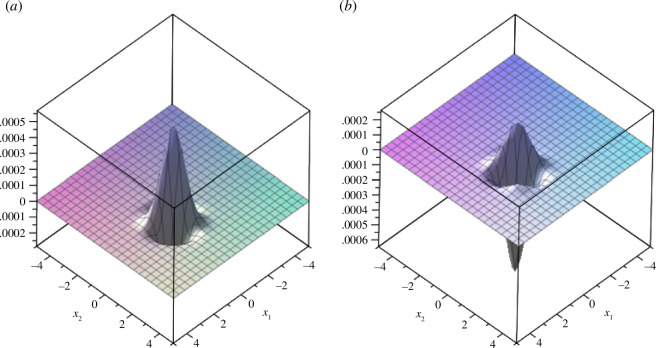
(*a*) Real part and (*b*) imaginary part of the coupled fractional Wigner–Ville distribution of example 3.5 in the spatial domain ( 𝒙 domain) for $\alpha =\frac{\pi }{4}$ , $\beta =\frac{\pi }{2}𝜼=5$ and 𝒙=[-5,5] .

**Figure 2.**
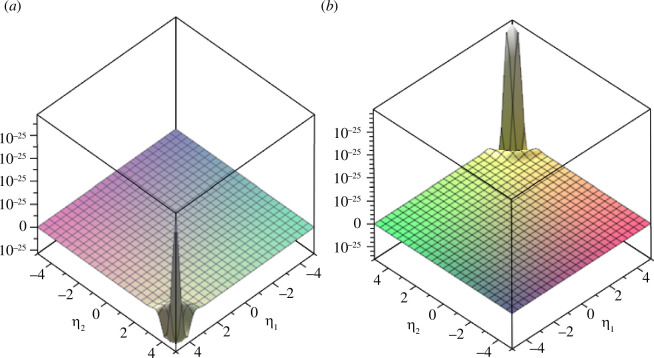
(*a*) Real part and (*b*) imaginary part of the coupled fractional Wigner–Ville distribution of example 3.5 in the frequency domain ( 𝜼 domain) for $\alpha =\frac{\pi }{4}$ , $\beta =\frac{\pi }{2}𝒙=5$ and 𝜼=[-5,5] .

We plot example 3.5 in figures 1 and 2.

**Example 3.6.** Find the CFrWVD of the functions f and g , defined by


(3.22) $$f(\tau )=g(\tau )=e^{-|\tau {|}^{2}}.$$

**Solution**. Substituting equation (3.22) into equation (2.18), we obtain


(3.23) $$\begin{array}{rl} W_{f,g}^{\alpha ,\beta }(x,\eta ) & =d(\gamma )\int_{\;\;R^{2}}e^{-{\left|x+\frac{\tau }{2}\right|}^{2}}e^{-{\left|x-\frac{\tau }{2}\right|}^{2}}e^{-i(a(\gamma )(|\tau {|}^{2}+|\eta {|}^{2})-\tau_{1}\left(b\eta_{1}+c\eta_{2}\right)-\tau_{2}\left(b\eta_{2}-c\eta_{1}\right))}d\tau_{1}d\tau_{2}\\ & =d(\gamma )\int_{\;\;R^{2}}e^{-\left({\left(x_{1}+\frac{\tau_{1}}{2}\right)}^{2}+{\left(x_{2}+\frac{\tau_{2}}{2}\right)}^{2}\right)}e^{-\left({\left(x_{1}-\frac{\tau_{1}}{2}\right)}^{2}+{\left(x_{2}-\frac{\tau_{2}}{2}\right)}^{2}\right)}\\ & \;\times e^{-i(a(\gamma )(\tau_{1}^{2}+\tau_{2}^{2}+\eta_{1}^{2}+\eta_{2}^{2})-\tau_{1}\left(b\eta_{1}+c\eta_{2}\right)-\tau_{2}\left(b\eta_{2}-c\eta_{1}\right)}d\tau_{1}d\tau_{2}\\ & =d(\gamma )\int_{\;\;R^{2}}e^{-\left({\left(x_{1}+\frac{\tau_{1}}{2}\right)}^{2}+{\left(x_{1}-\frac{\tau_{1}}{2}\right)}^{2}\right)}e^{-i(a(\gamma )\tau_{1}^{2}-\tau_{1}(b\eta_{1}+c\eta_{2}))}e^{-\left({\left(x_{2}+\frac{\tau_{2}}{2}\right)}^{2}+{\left(x_{2}-\frac{\tau_{2}}{2}\right)}^{2}\right)}\\ & \;\times e^{-i(a(\gamma )\tau_{2}^{2}-\tau_{2}(b\eta_{1}+c\eta_{2}))}e^{-ia(\gamma )(\eta_{1}^{2}+\eta_{2}^{2})}d\tau_{1}d\tau_{2}\\ & =d(\gamma )e^{-ia(\gamma )(\eta_{1}^{2}+\eta_{2}^{2})}\int_{\;\;R}e^{-\left(2\left(x_{1}^{2}+{\left(\frac{\tau_{1}}{2}\right)}^{2}\right)\right)-i(a(\gamma )\tau_{1}^{2}-\tau_{1}(b\eta_{1}+c\eta_{2}))}d\tau_{1}\\ & \;\times \int_{\;\;R}e^{-\left(2\left(x_{2}^{2}+{\left(\frac{\tau_{2}}{2}\right)}^{2}\right)\right)-i(a(\gamma )\tau_{2}^{2}-\tau_{1}(b\eta_{2}+c\eta_{1}))}d\tau_{2}. \end{array}$$

Furthermore, we get


(3.24) $$\begin{array}{rl} W_{f,g}^{\alpha ,\beta }(x,\eta )= & d(\gamma )e^{-ia(\gamma )(\eta_{1}^{2}+\eta_{2}^{2})-2(x_{1}^{2}+x_{2}^{2})}\int_{\;\;R^{2}}e^{-\frac{\tau_{1}^{2}}{2}-i(a(\gamma )\tau_{1}^{2}+\tau_{1}(b\eta_{1}+c\eta_{2}))}d\tau_{1}\\ & \;\times \int_{\;\;R^{2}}e^{-\frac{\tau_{2}^{2}}{2}-i(a(\gamma )\tau_{2}^{2}+\tau_{2}(b\eta_{2}-c\eta_{1}))}d\tau_{2}\\ = & d(\gamma )e^{-ia(\gamma )(\eta_{1}^{2}+\eta_{2}^{2})-2(x_{1}^{2}+x_{2}^{2})}\int_{\;\;R^{2}}e^{-\left(\frac{1}{2}+ia(\gamma )\right)\tau_{1}^{2}+\tau_{1}(b\eta_{1}+c\eta_{2})}d\tau_{1}\\ & \;\times \int_{\;\;R^{2}}e^{-\left(\frac{1}{2}+ia(\gamma )\right)\tau_{2}^{2}+\tau_{2}(b\eta_{2}-c\eta_{1})}d\tau_{2}. \end{array}$$

Equation (3.24) can be rewritten as


(3.25) $$\begin{array}{rl} W_{f,g}^{\alpha ,\beta }(x,\eta ) & =d(\gamma )e^{-ia(\gamma )(\eta_{1}^{2}+\eta_{2}^{2})-2(x_{1}^{2}+x_{2}^{2})}\int_{\;\;R}e^{-\left(\frac{1}{2}+ia(\gamma )\right)\left(\tau_{1}^{2}+\tau_{1}\frac{\left(b\eta_{1}+c\eta_{2}\right)}{\left(\frac{1}{2}+ia(\gamma )\right)}\right)}d\tau_{1}\\ & \;\times \int_{\;\;R}e^{-\left(\frac{1}{2}+ia(\gamma )\right)\left(\tau_{2}^{2}+\tau_{2}\frac{\left(b\eta_{2}-c\eta_{1}\right)}{\left(\frac{1}{2}+ia(\gamma )\right)}\right)}d\tau_{2}\\ & =d(\gamma )e^{-ia(\gamma )(\eta_{1}^{2}+\eta_{2}^{2})-2(x_{1}^{2}+x_{2}^{2})}\int_{\;\;R}e^{-\left(\frac{1}{2}+ia(\gamma )\right)\left({\left(\tau_{1}+\frac{\left(b\eta_{1}+c\eta_{2}\right)}{\left(1+i2a(\gamma )\right)}\right)}^{2}-{\left(\frac{\left(b\eta_{1}+c\eta_{2}\right)}{\left(1+i2a(\gamma )\right)}\right)}^{2}\right)}d\tau_{1}\\ & \;\times \int_{\;\;R}e^{-\left(\frac{1}{2}+ia(\gamma )\right)\left({\left(\tau_{2}+\frac{\left(b\eta_{2}-c\eta_{1}\right)}{\left(1+i2a(\gamma )\right)}\right)}^{2}-{\left(\frac{\left(b\eta_{2}-c\eta_{1}\right)}{\left(1+i2a(\gamma )\right)}\right)}^{2}\right)}d\tau_{2}\\ & =d(\gamma )e^{-ia(\gamma )(\eta_{1}^{2}+\eta_{2}^{2})-2(x_{1}^{2}+x_{2}^{2})+\left(\frac{1}{2}+ia(\gamma )\right){\left(\frac{\left(b\eta_{1}+c\eta_{2}\right)}{\left(1+i2a(\gamma )\right)}\right)}^{2}+\left(\frac{1}{2}+ia(\gamma )\right){\left(\frac{\left(b\eta_{2}-c\eta_{1}\right)}{\left(1+i2a(\gamma )\right)}\right)}^{2}}\\ & \;\times \int_{\;\;R}e^{-\left(\frac{1}{2}+ia(\gamma )\right){\left(\tau_{1}+\frac{b\eta_{1}+c\eta_{2}}{1+i2a(\gamma )}\right)}^{2}}d\tau_{1}\int_{\;\;R}e^{-\left(\frac{1}{2}+ia(\gamma )\right){\left(\tau_{2}+\frac{b\eta_{2}-c\eta_{1}}{1+i2a(\gamma )}\right)}^{2}}d\tau_{2}\\ & =d(\gamma )e^{-ia(\gamma )(\eta_{1}^{2}+\eta_{2}^{2})-2(x_{1}^{2}+x_{2}^{2})+\frac{(b\eta_{1}+c\eta_{2}{)}^{2}}{2(1+i2a(\gamma ))}+\frac{(b\eta_{2}-c\eta_{1}{)}^{2}}{2(1+i2a(\gamma ))}}\int_{\;\;R}e^{-{\left(\sqrt{\frac{1+i2a(\gamma )}{2}}\tau_{1}+\sqrt{2}\frac{\left(b\eta_{1}+c\eta_{2}\right)}{\sqrt{1+i2a(\gamma )}}\right)}^{2}}d\tau_{1}\\ & \;\times \int_{\;\;R}e^{-{\left(\sqrt{\frac{1+i2a(\gamma )}{2}}\tau_{2}+\sqrt{2}\frac{\left(b\eta_{2}-c\eta_{1}\right)}{\sqrt{1+i2a(\gamma )}}\right)}^{2}}d\tau_{2}. \end{array}$$

We finally arrive at


(3.26) $${𝒲}_{f,g}^{\alpha ,\beta }\,\left(𝒙,𝜼\right)=\frac{2\,\pi \,d\,\left(\gamma \right)}{1+i\,2\,a\,\left(\gamma \right)}\,e^{-i\,a\,\left(\gamma \right)\,\left(\eta_{1}^{2}+\eta_{2}^{2}\right)-2\,\left(x_{1}^{2}+x_{2}^{2}\right)+\frac{{\left(b\,\eta_{1}+c\,\eta_{2}\right)}^{2}+{\left(b\,\eta_{2}-c\,\eta_{1}\right)}^{2}}{2\,\left(1+i\,2\,a\,\left(\gamma \right)\right)}}.$$

**Figure 3.**
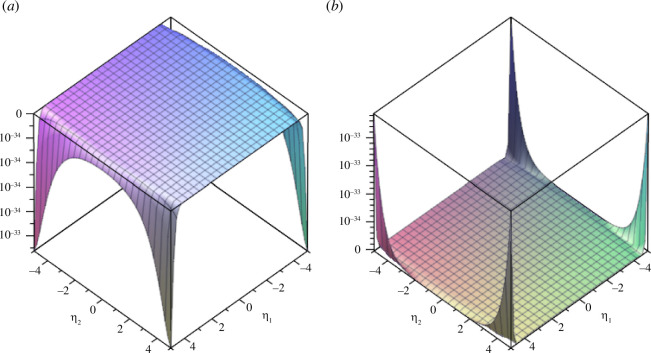
(*a*) Real part and (*b*) imaginary part of the coupled fractional Wigner–Ville distribution of example 3.6 in the frequency domain ( 𝜼 domain) for $\alpha =\frac{\pi }{4}$ , $\beta =\frac{\pi }{2}𝒙=5$ and 𝜼=[-5,5] .

**Figure 4.**
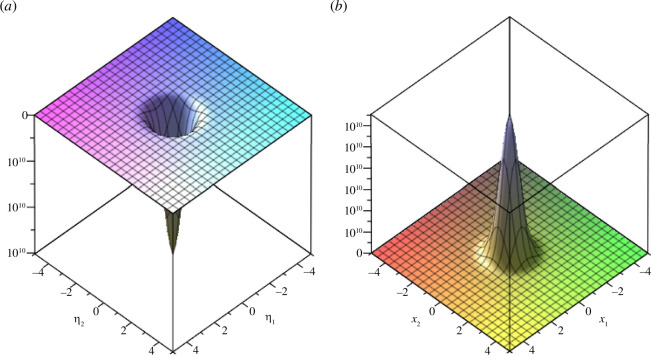
(*a*) Real part and (*b*) imaginary part of the coupled fractional Wigner–Ville distribution of example 3.6 in the spatial domain ( 𝒙 domain) for $\alpha =\frac{\pi }{4}$ , $\beta =\frac{\pi }{2}𝜼=5$ and 𝒙=[-5,5] .

We plot example 3.6 in figures 3 and 4.

## 4. Uncertainty principles for coupled fractional Wigner–Ville distribution

An uncertainty principle offers information about a signal and its Fourier transform in the time–frequency plane. More precisely, it states that a signal and its Fourier transform cannot simultaneously concentrate around a point. It is known that the most important property of any generalized transformation is the uncertainty principle. Therefore, various uncertainty principles of different types of transformations have been proposed [19–25]. In this section, we explore several versions of the uncertainty principles in the context of the CFrWVD.

### 4.1. Heisenberg uncertainty principle

Here, we shall expand the idea of the Heisenberg uncertainty principle for the two-dimensional Fourier transform to that of the CFrWVD. In this respect, we shall state and prove the following theorem.

**Theorem 4.1.** *Let two functions* $f,g\in L^{1}\,\left({\mathbb{R}}^{2}\right)\cap L^{2}\,\left({\mathbb{R}}^{2}\right)$ *. Then, one has*


(4.1) $$\begin{array}{r} \|f{\|}_{L^{2}(R^{2})}\leq \frac{16\pi^{2}}{|\sin\gamma {|}^{4}}\|g{\|}_{L^{2}(R^{2})}\int_{\;\;R^{2}}|\tau -x{|}^{2}|f(\tau ){|}^{2}d\tau \int_{R^{2}}\int_{\;\;R^{2}}|\eta {|}^{2}{\left|W_{f,g}^{\alpha ,\beta }\left(x,\eta \right)\right|}^{2}d\eta dx. \end{array}$$

*Proof*. By virtue of the uncertainty principle for the two-dimensional Fourier transform, we have


(4.2) $$\int_{\;\;R^{2}}{\left|\tau \right|}^{2}{\left|f\left(\tau \right)\right|}^{2}d\tau \int_{\;\;R^{2}}{\left|\eta \right|}^{2}|F\left\{f\right\}\left(\eta \right){|}^{2}d\eta \geq \frac{1}{4}{\left(\int_{\;\;R^{2}}{\left|f\left(\tau \right)\right|}^{2}d\tau \right)}^{\frac{1}{2}}.$$

Furthermore, we obtain


(4.3) $$\int_{\;\;R^{2}}{\left|\tau \right|}^{2}{\left|{\overset{ˇ}{h}}_{f,g}\left(x,\tau \right)\right|}^{2}d\tau \int_{\;\;R^{2}}{\left|\eta \right|}^{2}{\left|F\left\{{\overset{ˇ}{h}}_{f,g}\left(x,\tau \right)\right\}\left(\eta \right)\right|}^{2}d\eta \geq \frac{1}{4}{\left(\int_{\;\;R^{2}}{\left|{\overset{ˇ}{h}}_{f,g}\left(x,\tau \right)\right|}^{2}d\tau \right)}^{\frac{1}{2}}.$$

Substituting 𝜼 for -M⁢𝜼 in the above equation yields


(4.4) $$\begin{array}{rl} \int_{\;\;R^{2}}{\left|\tau \right|}^{2}{\left|{\overset{ˇ}{h}}_{f,g}\left(x,\tau \right)\right|}^{2}d\tau & \int_{\;\;R^{2}}{\left|\left(\det M\right)\right|}^{3}{\left|\eta \right|}^{2}{\left|F\left\{{\overset{ˇ}{h}}_{f,g}\left(x,\tau \right)\right\}\left(-M\eta \right)\right|}^{2}d\eta \\ & \geq \frac{1}{4}{\left(\int_{\;\;R^{2}}{\left|{\overset{ˇ}{h}}_{f,g}\left(x,\tau \right)\right|}^{2}d\tau \right)}^{\frac{1}{2}}. \end{array}$$

From equations (3.3) and (3.5) it will lead to


(4.5) $$\begin{array}{rl} |\det M{|}^{3}|d^{-1}(\gamma ){|}^{2} & \int_{\;\;R^{2}}{\left|\tau \right|}^{2}{\left|f\left(x+\frac{\tau }{2}\right)\bar{g\left(x-\frac{\tau }{2}\right)}\right|}^{2}d\tau \int_{\;\;R^{2}}{\left|\eta \right|}^{2}|e^{ia(\gamma )|\eta {|}^{2}}W_{f,g}^{\alpha ,\beta }(x,\eta ){|}^{2}d\eta \\ & \geq \frac{1}{4}{\left(\int_{\;\;R^{2}}{\left|f\left(x+\frac{\tau }{2}\right)\bar{g\left(x-\frac{\tau }{2}\right)}\right|}^{2}d\tau \right)}^{\frac{1}{2}}. \end{array}$$

Integrating both sides of equation ( 4.5 ) with respect to d⁢𝒙 , we obtain


(4.6) $$\begin{array}{rl} \frac{1}{4}\int_{\;\;R^{2}} & \int_{\;\;R^{2}}{\left|f\left(x+\frac{\tau }{2}\right)\bar{g\left(x-\frac{\tau }{2}\right)}\right|}^{2}d\tau dx\\ \leq & \frac{4\pi^{2}}{|\sin\gamma {|}^{4}}\int_{\;\;R^{2}}\int_{\;\;R^{2}}{\left|\tau \right|}^{2}{\left|f\left(x+\frac{\tau }{2}\right)\bar{g\left(x-\frac{\tau }{2}\right)}\right|}^{2}d\tau dx\int_{\;\;R^{2}}\int_{\;\;R^{2}}{\left|\eta \right|}^{2}{\left|W_{f,g}^{\alpha ,\beta }\left(x,\eta \right)\right|}^{2}d\eta dx. \end{array}$$

Fubini’s theorem gives


(4.7) $$\begin{array}{rl} & {\left(\int_{\;\;R^{2}}{\left|f\left(\tau \right)\right|}^{2}d\tau \right)}^{\frac{1}{2}}{\left(\int_{\;\;R^{2}}{\bar{\left|g\left(x\right)\right|}}^{2}dx\right)}^{\frac{1}{2}}\\ \leq & \frac{16\pi^{2}}{|\sin\gamma {|}^{4}}\int_{\;\;R^{2}}{\left|2\left(\tau -x\right)\right|}^{2}{\left|f\left(\tau \right)\right|}^{2}d\tau \int_{\;\;R^{2}}{\left|g\left(x\right)\right|}^{2}dx\int_{\;\;R^{2}}\int_{\;\;R^{2}}{\left|\eta \right|}^{2}{\left|W_{f,g}^{\alpha ,\beta }\left(x,\eta \right)\right|}^{2}d\eta dx. \end{array}$$

Hence,


$$\begin{array}{r} \|f{\|}_{L^{2}(\;\;R^{2})}\leq \frac{16\pi^{2}}{|\sin\gamma {|}^{4}}\|g{\|}_{L^{2}(\;\;R^{2})}\int_{\;\;R^{2}}|\tau -x{|}^{2}|f(\tau ){|}^{2}d\tau \int_{\;\;R^{2}}\int_{\;\;R^{2}}|\eta {|}^{2}{\left|W_{f,g}^{\alpha ,\beta }\left(x,\eta \right)\right|}^{2}d\eta dx, \end{array}$$

which proves the theorem.

### 4.2. Sharp Hausdorff–Young inequality

The purpose of this part is to build sharp Hausdorff–Young inequality related to the CFrWVD. This principle generalizes sharp Hausdorff–Young inequality for the two-dimensional Fourier transform to the CFrWVD. This principle is very useful in deriving Lieb’s inequality related to the proposed CFrWVD.

**Theorem 4.2.** *Let* p∈[1,2] *, such that* $\frac{1}{p}+\frac{1}{q}=1$ *, then for any* $f,g\in L^{2}\,\left({\mathbb{R}}^{2}\right)$ *, there holds*


(4.8) $${\left(\int_{\;\;R^{2}}\int_{\;\;R^{2}}\left|W_{f,g}^{\alpha ,\beta }{\left(x,\eta \right)}^{p}d\eta dx\right|\right)}^{\frac{1}{p}}\leq \frac{4^{\frac{1}{q}}sin\;{\left|\gamma \right|}^{\frac{2}{p}-1}}{2\pi }C(p){\left\|f\right\|}_{L^{q}\left(R^{2}\right)}{\left\|g\right\|}_{L^{q}\left(R^{2}\right)},$$

*where*


(4.9) $$C\,\left(p\right)=p^{\frac{1}{p}}\,q^{-\frac{1}{q}}.$$

*Proof*. By virtue of sharp Hausdorff–Young inequality for the two-dimensional Fourier transform, it follows that


(4.10) $${\left(\int_{\;\;R^{2}}{\left|F\left\{f\right\}\left(\eta \right)\right|}^{p}d\eta \right)}^{\frac{1}{p}}\leq C\left(p\right){\left(\int_{\;\;R^{2}}{\left|f\left(\tau \right)\right|}^{q}d\tau \right)}^{\frac{1}{q}}.$$

Inserting f⁢(𝝉) by ${\overset{ˇ}{h}}_{f,g}\,\left(𝒙,𝝉\right)$ to both sides of equation (4.10) results in


(4.11) $${\left(\int_{\;\;R^{2}}{\left|F\left\{{\overset{ˇ}{h}}_{f,g}\left(x,\tau \right)\left(\eta \right)\right\}\right|}^{p}d\eta \right)}^{\frac{1}{p}}\leq C\left(p\right){\left(\int_{\;\;R^{2}}{\left|{\overset{ˇ}{h}}_{f,g}\left(x,\tau \right)\right|}^{q}d\tau \right)}^{\frac{1}{q}}.$$

Substituting 𝜼 for -M⁢𝜼 in equation (4.11), it is turned into


(4.12) $$\begin{array}{rl} (\int_{\;\;R^{2}} & {\left|F\left\{{\overset{ˇ}{h}}_{f,g}\left(x,\tau \right)\left(-M\eta \right)\right\}\right|}^{p}d\left(-M\eta \right){)}^{\frac{1}{p}}\\ & \leq C\left(p\right){\left(\int_{\;\;R^{2}}{\left|f\left(x+\frac{\tau }{2}\right)\bar{g\left(x-\frac{\tau }{2}\right)}e^{-ia(\gamma )|\tau {|}^{2}}\right|}^{q}d\tau \right)}^{\frac{1}{q}}. \end{array}$$

Due to equation (3.3), we obtain


(4.13) $$\frac{2\pi |\sin\gamma |}{|\sin\gamma {|}^{\frac{2}{p}}}{\left(\int_{\;\;R^{2}}{\left|W_{f,g}^{\alpha ,\beta }\left(x,\eta \right)\right|}^{p}d\eta \right)}^{\frac{1}{p}}\leq C\left(p\right){\left(\int_{\;\;R^{2}}{\left|f\left(x+\frac{\tau }{2}\right)\bar{g\left(x-\frac{\tau }{2}\right)}\right|}^{q}d\tau \right)}^{\frac{1}{q}}.$$

If we integrate equation (4.13) with respect to d⁢𝒙 , then we get


(4.14) $$\begin{array}{r} \frac{2\pi |\sin\gamma |}{|\sin\gamma {|}^{\frac{2}{p}}}{\left(\int_{\;\;R^{2}}\int_{\;\;R^{2}}{\left|W_{f,g}^{\alpha ,\beta }\left(x,\eta \right)\right|}^{p}d\eta dx\right)}^{\frac{1}{p}}\leq C\left(p\right){\left(\int_{\;\;R^{2}}\int_{\;\;R^{2}}{\left|f\left(x+\frac{\tau }{2}\right)\bar{g\left(x-\frac{\tau }{2}\right)}\right|}^{q}d\tau dx\right)}^{\frac{1}{q}}. \end{array}$$

This equation is the same as


(4.15) $$\begin{array}{r} 2\pi |\sin\gamma {|}^{1-\frac{2}{p}}{\left(\int_{\;\;R^{2}}\int_{\;\;R^{2}}{\left|W_{f,g}^{\alpha ,\beta }\left(x,\eta \right)\right|}^{p}d\eta dx\right)}^{\frac{1}{p}}\leq 4^{\frac{1}{q}}C\left(p\right){\left(\int_{\;\;R^{2}}{\left|f\left(\tau \right)\right|}^{q}d\tau \right)}^{\frac{1}{q}}{\left(\int_{\;\;R^{2}}{\left|g\left(x\right)\right|}^{q}dx\right)}^{\frac{1}{q}}. \end{array}$$

Furthermore,


$$\begin{array}{r} {\left(\int_{\;\;R^{2}}\int_{\;\;R^{2}}{\left|W_{f,g}^{\alpha ,\beta }\left(x,\eta \right)\right|}^{p}d\eta dx\right)}^{\frac{1}{p}}\leq \frac{4^{\frac{1}{q}}\sin|\gamma {|}^{\frac{2}{p}-1}}{2\pi }C\left(p\right){\left\|f\right\|}_{L^{q}\left(R^{2}\right)}{\left\|g\right\|}_{L^{q}\left(R^{2}\right)}, \end{array}$$

and the proof is complete. ∎

### 4.3. Lieb’s inequality

Lieb’s inequality can be generalized to the coupled Wigner–Ville distribution case. Below, we use sharp Hausdorff–Young inequality mentioned earlier to prove Lieb’s inequality concerning the coupled Wigner–Ville distribution. To this interest, we obtain the following important result.

**Theorem 4.3.** *For two functions* $f,g\in L^{2}\,\left({\mathbb{R}}^{2}\right)$ *and* 2≤p§lt;∞ *, one has*


(4.16) $$\begin{array}{r} {\left(\int_{\;\;R^{2}}\int_{\;\;R^{2}}{\left|W_{f,g}^{\alpha ,\beta }\left(x,\eta \right)\right|}^{p}d\eta dx\right)}^{\frac{1}{p}}\leq \frac{4^{\frac{1}{q}-\frac{1}{p}+2}}{q^{2}2\pi }{\left|\sin\gamma \right|}^{\frac{2}{p}-1}{\left(\frac{2}{q}\right)}^{-\frac{2}{p}}{\left\|f\right\|}_{L^{2}\left(R^{2}\right)}{\left\|g\right\|}_{L^{2}\left(R^{2}\right)}, \end{array}$$

*where* $\frac{1}{p}+\frac{1}{q}=1$ .

*Proof*. From equation (4.13), it follows that


(4.17) $$\begin{array}{rl} {\left(\int_{\;\;R^{2}}{\left|W_{f,g}^{\alpha ,\beta }\left(x,\eta \right)\right|}^{p}d\eta \right)}^{\frac{1}{p}} & \leq \frac{|\sin\gamma {|}^{\frac{2}{p}-1}}{2\pi }C\left(p\right){\left(\int_{\;\;R^{2}}{\left|f\left(x+\frac{\tau }{2}\right)\bar{g\left(x-\frac{\tau }{2}\right)}\right|}^{q}d\tau \right)}^{\frac{1}{q}}. \end{array}$$

Using the substitution $𝒚=𝒙+\frac{𝝉}{2}$ , we obtain


(4.18) $$\begin{array}{r} {\left(\int_{\;\;R^{2}}{\left|W_{f,g}^{\alpha ,\beta }\left(x,\eta \right)\right|}^{p}d\eta \right)}^{\frac{1}{p}}\leq \frac{4^{\frac{1}{q}}|\sin\gamma {|}^{\frac{2}{p}-1}}{2\pi }C\left(p\right){\left(\int_{\;\;R^{2}}{\left|f\left(y\right)\bar{g\left(2x-y\right)}\right|}^{q}dy\right)}^{\frac{1}{q}}, \end{array}$$

or


(4.19) $$\begin{array}{r} \int_{\;\;R^{2}}{\left|W_{f,g}^{\alpha ,\beta }\left(x,\eta \right)\right|}^{p}d\eta \leq \frac{4^{\frac{p}{q}}|\sin\gamma {|}^{(\frac{2}{p}-1)p}}{{\left(2\pi \right)}^{p}}C{\left(p\right)}^{p}{\left(\int_{\;\;R^{2}}{\left|f\left(y\right)\bar{g\left(2x-y\right)}\right|}^{q}dy\right)}^{\frac{p}{q}}. \end{array}$$

We integrate both sides of equation (4.19) with respect to d⁢𝒙 and obtain


(4.20) $$\int_{\;\;R^{2}}\int_{\;\;R^{2}}{\left|W_{f,g}^{\alpha ,\beta }\left(x,\eta \right)\right|}^{p}d\eta dx\leq \frac{4^{\frac{p}{q}}|\sin\gamma {|}^{(\frac{2}{p}-1)p}}{{\left(2\pi \right)}^{p}}C{\left(p\right)}^{p}{\left(\int_{\;\;R^{2}}\int_{\;\;R^{2}}{\left|f\left(y\right)\bar{g\left(2x-y\right)}\right|}^{q}dy\right)}^{\frac{p}{q}}dx.$$

Using the change of the variables, 𝒖=2⁢𝒙 gives


(4.21) $$\begin{array}{r} {\left(\int_{\;\;R^{2}}\int_{\;\;R^{2}}{\left|W_{f,g}^{\alpha ,\beta }\left(x,\eta \right)\right|}^{p}d\eta dx\right)}^{\frac{1}{p}}\leq \frac{4^{\frac{1}{q}}{\left|\sin\gamma \right|}^{\frac{2}{p}-1}}{2\pi }C\left(p\right){\left(\int_{\;\;R^{2}}{\left(\int_{\;\;R^{2}}{\left|f\left(y\right)\bar{g\left(u-y\right)}\right|}^{q}dy\right)}^{\frac{p}{q}}\frac{du}{4}\right)}^{\frac{1}{p}}. \end{array}$$

Hence,


(4.22) $$\begin{array}{r} {\left(\int_{\;\;R^{2}}\int_{\;\;R^{2}}{\left|W_{f,g}^{\alpha ,\beta }\left(x,\eta \right)\right|}^{p}d\eta dx\right)}^{\frac{1}{p}}\leq \frac{4^{\frac{1}{q}-\frac{1}{p}}{\left|\sin\gamma \right|}^{\frac{2}{p}-1}}{2\pi }C\left(p\right){\left(\int_{\;\;R^{2}}{\left(\int_{\;\;R^{2}}{\left|f\left(y\right)\bar{g\left(u-y\right)}\right|}^{q}dy\right)}^{\frac{p}{q}}du\right)}^{\frac{1}{\frac{p}{q}}\frac{1}{q}}. \end{array}$$

Equation (4.22) may be expressed as


(4.23) $$\begin{array}{r} {\left(\int_{\;\;R^{2}}\int_{\;\;R^{2}}{\left|W_{f,g}^{\alpha ,\beta }\left(x,\eta \right)\right|}^{p}d\eta dx\right)}^{\frac{1}{p}}\leq \frac{4^{\frac{1}{q}-\frac{1}{p}}{\left|\sin\gamma \right|}^{\frac{2}{p}-1}}{2\pi }C\left(p\right){\left({\left(\int_{\;\;R^{2}}{\left(\left({\left|f\right|}^{q}*{\left|g\right|}^{q}\right)\left(u\right)\right)}^{\frac{p}{q}}du\right)}^{\frac{1}{\frac{p}{q}}}\right)}^{\frac{1}{q}}. \end{array}$$

We write relation (4.23) as


(4.24) $$\begin{array}{r} {\left(\int_{\;\;R^{2}}\int_{\;\;R^{2}}{\left|W_{f,g}^{\alpha ,\beta }\left(x,\eta \right)\right|}^{p}d\eta dx\right)}^{\frac{1}{p}}\leq \frac{4^{\frac{1}{q}-\frac{1}{p}}{\left|\sin\gamma \right|}^{\frac{2}{p}-1}}{2\pi }C\left(p\right){\left\|{\left|f\right|}^{q}*{\left|g\right|}^{q}\right\|}_{L^{\frac{p}{q}}\left(R^{2}\right)}^{\frac{1}{q}}. \end{array}$$

Since $f,g\in L^{2}\,\left({\mathbb{R}}^{2}\right)$ then ${\left|f\right|}^{q}$ , ${\left|g\right|}^{q}\in L^{2}\,\left({\mathbb{R}}^{2}\right)$ . Applying the Young inequality for ${\left|f\right|}^{q}$ and ${\left|g\right|}^{q}$ with triple $\left(p^{\prime },p^{\prime },t\right)=\left(\frac{2}{q},\frac{2}{q},\frac{p}{q}\right)$ , we obtain


(4.25) $$\begin{array}{cc} {∥{\left|f\right|}^{q}*{\left|g\right|}^{q}∥}_{L^{t}\,\left({\mathbb{R}}^{2}\right)}\leq C\,{\left(p\right)}^{\frac{4}{q}}\,C\,{\left(t\right)}^{\frac{2}{q}}\,{∥{\left|f\right|}^{q}∥}_{L^{p^{\prime }}\,\left({\mathbb{R}}^{2}\right)}\,{∥{\left|g\right|}^{q}∥}_{L^{p^{\prime }}\,\left({\mathbb{R}}^{2}\right)}. & \end{array}$$

Observe that


(4.26) $$\begin{array}{cc} C\,\left(p\right)\,C\,{\left(p\right)}^{\frac{4}{r}}\,C\,{\left(t\right)}^{\frac{2}{r}} & =\left(\frac{2}{q^{1+\frac{1}{q}-\frac{1}{p}}}\right)\,{\left(\frac{1}{2}\right)}^{\frac{q-p}{p\,q}}\\ & =\frac{4}{q^{2}}\,{\left(\frac{2}{q}\right)}^{-\frac{2}{p}}. \end{array}$$

Substituting equation (4.26) into equation (4.24) gives


$$\begin{array}{r} {\left(\int_{\;\;R^{2}}\int_{\;\;R^{2}}{\left|W_{f,g}^{\alpha ,\beta }\left(x,\eta \right)\right|}^{p}d\eta dx\right)}^{\frac{1}{p}}\leq \frac{4^{\frac{1}{q}-\frac{1}{p}+2}}{2\pi q^{2}}{\left|\sin\gamma \right|}^{\frac{2}{p}-1}{\left(\frac{2}{q}\right)}^{-\frac{2}{p}}\|f{\|}_{L^{2}(R^{2})}\|g{\|}_{L^{2}(R^{2})}, \end{array}$$

which finishes the proof.

### 4.4. Logarithmic Sobolev-type inequality

In this section, we formulate a Sobolev-type inequality for the CFrWVD. To carry our endeavour, we shall provide some basic definitions.

**Definition 4.4.** Given the operator $𝒟=\left(\frac{\partial }{\partial t_{1}},\frac{\partial }{\partial t_{2}}\right)$ , the Sobolev space $𝒮\,\left({\mathbb{R}}^{2}\right)$ on ${\mathbb{R}}^{2}$ is defined as


(4.27) $$S(R^{2})=\left\{f\in L^{2}\left(R^{2}\right);D_{f}\in L^{2}\left(R^{2}\right)\right\}.$$

**Definition 4.5.** For *1* ≤p<∞ and j>0 , the weighted Lebesgue space ${𝒲}_{j}^{p}\,\left({\mathbb{R}}^{2}\right)$ on ${\mathbb{R}}^{2}$ is defined by


(4.28) $${𝒲}_{j}^{p}\,\left({\mathbb{R}}^{2}\right)=\left\{f\in L_{l\,o\,c}^{p}\,\left({\mathbb{R}}^{2}\right):{\left\langle 𝒙\right\rangle }^{j}\,f\in L^{p}\,\left({\mathbb{R}}^{2}\right)\right\},$$

where $\left\langle 𝐱\right\rangle ={\left(1+{\left|𝐱\right|}^{2}\right)}^{\frac{1}{2}}$ is the weight function.

Let us derive the following result.

**Theorem 4.6.** *Let two functions* $f,g\in 𝒮\,\left({\mathbb{R}}^{2}\right)\cap {𝒲}_{j}^{p}\,\left({\mathbb{R}}^{2}\right)$ *, and the following inequality be satisfied:*


(4.29) $$\begin{array}{rl} {\left\|f\right\|}_{L^{2}\left(R^{2}\right)}^{2}\int_{\;\;R^{2}}{\left|\bar{g\left(x-\frac{\tau }{2}\right)}\right|}^{2} & \ln\left(\frac{1+{\left|\tau \right|}^{2}}{2}\right)d\tau \\ & +4\pi^{2}\int_{\;\;R^{2}}\int_{\;\;R^{2}}\left(\ln\left|\frac{1}{\sin^{4}\gamma }\right|+\ln{\left|\eta \right|}^{2}\right){\left|W_{f,g}^{\alpha ,\beta }(x,\eta )\right|}^{2}d\eta dx\\ & \geq \frac{4\Gamma^{\prime }\left(1\right)}{\Gamma \left(1\right)}\|f{\|}_{L^{2}(R^{2})}^{2}\|g{\|}_{L^{2}(R^{2})}^{2}, \end{array}$$

*where* Γ⁢(⋅) *is gamma function.*

*Proof*. With the help of logarithmic Sobolev-type inequality for the two-dimensional Fourier transform, we have


(4.30) $$\int_{\;\;R^{2}}{\left|f\left(\tau \right)\right|}^{2}\ln\left(\frac{1+{\left|\tau \right|}^{2}}{2}\right)d\tau +\int_{\;\;R^{2}}\ln{\left|\eta \right|}^{2}{\left|F\left\{f\right\}\left(\eta \right)\right|}^{2}d\eta \geq \frac{\Gamma^{\prime }\left(1\right)}{\Gamma \left(1\right)}\int_{R^{2}}{\left|f\left(\tau \right)\right|}^{2}d\tau .$$

Inserting f⁢(𝝉) with ${\overset{ˇ}{h}}_{f,g}\,\left(𝒙,𝝉\right)$ and then setting 𝜼 with -M⁢𝜼 results in


(4.31) $$\begin{array}{rl} \int_{\;\;R^{2}}{\left|{\overset{ˇ}{h}}_{f,g}(x,\tau )\right|}^{2}\ln\left(\frac{1+{\left|\tau \right|}^{2}}{2}\right)d\tau + & \ln|\eta {|}^{2}\int_{\;\;R^{2}}{\left|F\left\{{\overset{ˇ}{h}}_{f,g}\left(x,\tau \right)\right\}\left(\eta \right)\right|}^{2}d\eta \\ & \geq \frac{\Gamma^{\prime }\left(1\right)}{\Gamma \left(1\right)}\int_{\;\;R^{2}}{\left|{\overset{ˇ}{h}}_{f,g}\left(x,\tau \right)\right|}^{2}d\tau . \end{array}$$

Hence,


(4.32) $$\begin{array}{rl} \int_{\;\;R^{2}}|{\overset{ˇ}{h}}_{f,g}( & x,\tau ){|}^{2}\ln\left(\frac{1+{\left|\tau \right|}^{2}}{2}\right)d\tau +\ln{\left|\det M\right|}^{2}\ln{\left|\eta \right|}^{2}\\ & \times \int_{\;\;R^{2}}{\left|F\left\{{\overset{ˇ}{h}}_{f,g}\left(x,\tau \right)\right\}\left(-M\eta \right)\right|}^{2}|\det M|d\eta \geq \frac{\Gamma^{\prime }\left(1\right)}{\Gamma \left(1\right)}\int_{\;\;R^{2}}{\left|{\overset{ˇ}{h}}_{f,g}\left(x,\tau \right)\right|}^{2}d\tau . \end{array}$$

On application of equations (3.3) and (3.5) to equation (4.32), we get


(4.33) $$\begin{array}{rl} \int_{\;\;R^{2}}|f\left(x+\frac{\tau }{2}\right) & \bar{g\left(x-\frac{\tau }{2}\right)}e^{-ia(\gamma )|\tau {|}^{2}}{|}^{2}\ln\left(\frac{1+{\left|\tau \right|}^{2}}{2}\right)d\tau \\ & +\int_{\;\;R^{2}}\left(\ln\left|\frac{1}{\sin^{4}\gamma }\right|+\ln{\left|\eta \right|}^{2}\right){\left|d^{-1}(\gamma )e^{ia(\gamma )|\eta {|}^{2}}W_{f,g}^{\alpha ,\beta }(x,\eta )\right|}^{2}\left(\frac{1}{\sin^{2}\gamma }\right)d\eta \\ & \geq \frac{\Gamma^{\prime }\left(1\right)}{\Gamma \left(1\right)}\int_{\;\;R^{2}}{\left|f\left(x+\frac{\tau }{2}\right)\bar{g\left(x-\frac{\tau }{2}\right)}e^{-ia(\gamma )|\tau {|}^{2}}\right|}^{2}d\tau . \end{array}$$

Equation (4.33) can be rewritten as


(4.34) $$\begin{array}{rl} \int_{\;\;R^{2}}|f\left(x+\frac{\tau }{2}\right) & \bar{g\left(x-\frac{\tau }{2}\right)}{|}^{2}\ln\left(\frac{1+{\left|\tau \right|}^{2}}{2}\right)d\tau \\ & +{\left|d^{-1}(\gamma )\right|}^{2}\int_{\;\;R^{2}}\left(\ln\left|\frac{1}{\sin^{4}\gamma }\right|+\ln{\left|\eta \right|}^{2}\right){\left|W_{f,g}^{\alpha ,\beta }(x,\eta )\right|}^{2}\left(\frac{1}{\sin^{2}\gamma }\right)d\eta \\ & \geq \frac{\Gamma^{\prime }\left(1\right)}{\Gamma \left(1\right)}\int_{\;\;R^{2}}{\left|f\left(x+\frac{\tau }{2}\right)\bar{g\left(x-\frac{\tau }{2}\right)}\right|}^{2}d\tau . \end{array}$$

This equation is equal to


(4.35) $$\begin{array}{rl} \int_{\;\;R^{2}}|f\left(x+\frac{\tau }{2}\right) & \bar{g\left(x-\frac{\tau }{2}\right)}{|}^{2}\ln\left(\frac{1+{\left|\tau \right|}^{2}}{2}\right)d\tau \\ & +4\pi^{2}\sin^{2}\gamma \int_{\;\;R^{2}}\left(\ln\left|\frac{1}{\sin^{4}\gamma }\right|+\ln{\left|\eta \right|}^{2}\right){\left|W_{f,g}^{\alpha ,\beta }(x,\eta )\right|}^{2}\left(\frac{1}{\sin^{2}\gamma }\right)d\eta \\ & \geq \frac{\Gamma^{\prime }\left(1\right)}{\Gamma \left(1\right)}\int_{\;\;R^{2}}{\left|f\left(x+\frac{\tau }{2}\right)\bar{g\left(x-\frac{\tau }{2}\right)}\right|}^{2}d\tau . \end{array}$$

Integrating both sides of equation (4.35) with respect to d⁢𝒙 , we have


(4.36) $$\begin{array}{rl} \int_{\;\;R^{2}}\int_{R^{2}}|f\left(x+\frac{\tau }{2}\right) & \bar{g\left(x-\frac{\tau }{2}\right)}{|}^{2}\ln\left(\frac{1+{\left|\tau \right|}^{2}}{2}\right)dxd\tau \\ & +4\pi^{2}\sin^{2}\gamma \int_{\;\;R^{2}}\int_{\;\;R^{2}}\ln\left|\frac{1}{\sin^{4}\gamma }\right|{\left|W_{f,g}^{\alpha ,\beta }(x,\eta )\right|}^{2}\left(\frac{1}{\sin^{2}\gamma }\right)d\eta dx\\ & +4\pi^{2}\sin^{2}\gamma \int_{\;\;R^{2}}\int_{\;\;R^{2}}\ln{\left|\eta \right|}^{2}{\left|W_{f,g}^{\alpha ,\beta }(x,\eta )\right|}^{2}\left(\frac{1}{\sin^{2}\gamma }\right)d\eta dx\\ & \geq \frac{\Gamma^{\prime }\left(1\right)}{\Gamma \left(1\right)}\int_{\;\;R^{2}}\int_{\;\;R^{2}}{\left|f\left(x+\frac{\tau }{2}\right)\bar{g\left(x-\frac{\tau }{2}\right)}\right|}^{2}dxd\tau . \end{array}$$

Equation (4.36) can be expressed as


(4.37) $$\begin{array}{rl} \int_{\;\;R^{2}}{\left|f\left(x+\frac{\tau }{2}\right)\right|}^{2}dx & \int_{\;\;R^{2}}{\left|\bar{g\left(x-\frac{\tau }{2}\right)}\right|}^{2}\ln\left(\frac{1+{\left|\tau \right|}^{2}}{2}\right)d\tau \\ & +4\pi^{2}\sin^{2}\gamma \int_{\;\;R^{2}}\int_{\;\;R^{2}}\ln\left|\frac{1}{\sin^{4}\gamma }\right|{\left|W_{f,g}^{\alpha ,\beta }(x,\eta )\right|}^{2}\left(\frac{1}{\sin^{2}\gamma }\right)d\eta dx\\ & +4\pi^{2}\sin^{2}\gamma \int_{\;\;R^{2}}\int_{\;\;R^{2}}\ln{\left|\eta \right|}^{2}{\left|W_{f,g}^{\alpha ,\beta }(x,\eta )\right|}^{2}\left(\frac{1}{\sin^{2}\gamma }\right)d\eta dx\\ & \geq \frac{\Gamma^{\prime }\left(1\right)}{\Gamma \left(1\right)}\int_{\;\;R^{2}}{\left|f\left(x+\frac{\tau }{2}\right)\right|}^{2}dx\int_{\;\;R^{2}}{\left|\bar{g\left(x-\frac{\tau }{2}\right)}\right|}^{2}d\tau . \end{array}$$

Equation (4.37) can be rewritten in the form


$$\begin{array}{rl} {\left\|f\right\|}_{L^{2}\left(\;\;R^{2}\right)}^{2}\int_{R^{2}}{\left|\bar{g\left(x-\frac{\tau }{2}\right)}\right|}^{2} & \ln\left(\frac{1+{\left|\;\;\tau \right|}^{2}}{2}\right)d\tau \\ & +4\pi^{2}\int_{\;\;R^{2}}\int_{\;\;R^{2}}\ln\left|\frac{1}{\sin^{4}\gamma }\right|{\left|W_{f,g}^{\alpha ,\beta }(x,\eta )\right|}^{2}d\eta dx\\ & +4\pi^{2}\int_{\;\;R^{2}}\int_{\;\;R^{2}}\ln{\left|\eta \right|}^{2}{\left|W_{f,g}^{\alpha ,\beta }(x,\eta )\right|}^{2}d\eta dx\\ & \geq \frac{4\Gamma^{\prime }\left(1\right)}{\Gamma \left(1\right)}\|f{\|}_{L^{2}(R^{2})}^{2}\|g{\|}_{L^{2}(R^{2})}^{2}. \end{array}$$

This equation is equal to


$$\begin{array}{rl} {\left\|f\right\|}_{L^{2}\left(R^{2}\right)}^{2}\int_{R^{2}}{\left|\bar{g\left(x-\frac{\tau }{2}\right)}\right|}^{2} & \ln\left(\frac{1+{\left|\tau \right|}^{2}}{2}\right)d\tau \\ & +4\pi^{2}\int_{\;\;R^{2}}\int_{\;\;R^{2}}\left(\ln\left|\frac{1}{\sin^{4}\gamma }\right|+\ln{\left|\eta \right|}^{2}\right){\left|W_{f,g}^{\alpha ,\beta }(x,\eta )\right|}^{2}d\eta dx\\ & \geq \frac{4\Gamma^{\prime }\left(1\right)}{\Gamma \left(1\right)}\|f{\|}_{L^{2}(R^{2})}^{2}\|g{\|}_{L^{2}(R^{2})}^{2}, \end{array}$$

which completes the proof.

**Remark 4.7.** The authors of [15] have presented several uncertainty principles related to CFrWVD such as Hardy’s and Beurling’s uncertainty inequalities which were not investigated in this article. The proof of their uncertainty principles used the definition of the CFrWVD and its properties, while our work is derived by developing the basic relationship between the CFrWVD and the Fourier transform.

## 5. Conclusion

In this article, we have introduced the CFrWVD and investigated its properties. Also, we presented the close link between the CFrWVD and the Fourier transform. We combined this relation and properties of the CFrWVD to see for several versions of the uncertainty principles related to the proposed transformation. The uncertainty inequalities play a key role in understanding and development of signal analysis.

## Data Availability

This article has no additional data.
